# Epilepsy Caused by Neurocysticercosis: A Case Report

**DOI:** 10.21980/J81P96

**Published:** 2023-01-31

**Authors:** Mary G McGoldrick, Daniel Polvino, Grant Wei

**Affiliations:** * Rutgers Robert Wood Johnson Medical School, Department of Emergency Medicine, New Brunswick, NJ

## Abstract

Neurocysticercosis is one of the most common causes of acquired epilepsy worldwide. This diagnosis is rarely considered in patients presenting to the emergency department (ED) with recurrent seizures in the United States (U.S.). This is the case of a young adult male presenting with post-ictal confusion and recurrent seizures requiring intubation for agitation and airway protection. It illustrates the need to maintain a broad differential when considering etiology of seizures in a globalized patient population.

**Topics:**

Seizure, neurocysticercosis, epilepsy, parasitic infection.

**Figure f1-jetem-8-1-v14:**
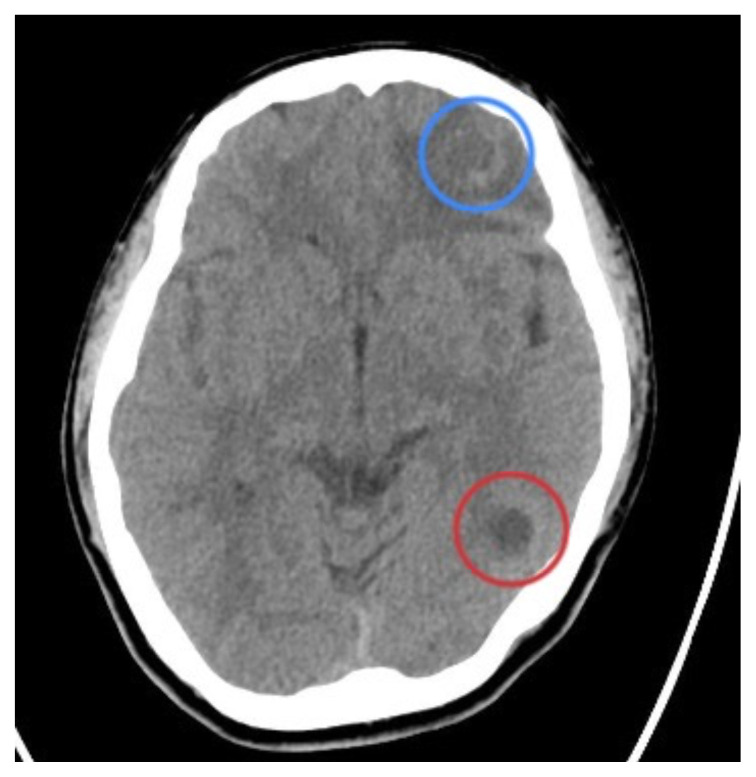


## Brief introduction

[Fig f1-jetem-8-1-v14]Neurocysticercosis (NCC) is a central nervous system (CNS) infection caused by ingestion of *Taenia soleum* larvae, most commonly via infected pork in regions of the world with poor regulation of food safety and sanitation.^1^ The primary manifestation of the disease in greater than 60% of cases is intraparenchymal neurocysticercosis, resulting in cyst development and subsequent degeneration in the brain parenchyma. Patients can become symptomatic months to years following the initial ingestion as the cysts degenerate, resulting in focal or generalized seizures. Endemic areas include Asia, Africa, and South America, where it is a leading cause of acquired epilepsy.^2^ While not endemic to the United States, NCC is responsible for an estimated 1000 hospital presentations per year, recognized by the CDC as a “neglected parasitic infection.”^3^ Diagnosis is dependent on multiple factors including symptoms, imaging with computed tomography (CT) and magnetic resonance imaging (MRI), and serology. Treatment recommendations vary depending on the severity and stage of the disease, but frequently employ antiparasitic agents and antiepileptic drugs.^4^ Recognition of the symptoms in patients from endemic areas is key, and emergency medicine (EM) clinicians could play a large role in reducing morbidity and mortality from this preventable cause of epilepsy. Characteristic neuroimaging is presented in the hopes of increasing consideration of this disease process when evaluating patients for new-onset seizure.

## Presenting concerns and clinical findings

The patient was a 24-year-old Hispanic male with unclear past medical history, possibly significant for seizures, who presented with emergency medical services (EMS) after a described five minutes of tonic-clonic seizure activity which was witnessed by family. Medics reported that the patient had told family about a fall at work the day prior. According to the family, the patient may have had seizures in the past but was not on medication. The seizure resolved prior to EMS arrival, and on initial EMS presentation the patient was confused, oriented only to himself, and only intermittently followed commands.

On arrival to the ED, the patient was alert and oriented to person, place, time, and answering questions, but was unclear on the events leading up to presentation. He stated that he fell at work and hit his head without loss of consciousness, and that he had been drinking beer the night prior. He denied any history of seizures and endorsed weekly alcohol use, but no history of withdrawal. His only complaint was of pain in his forehead and generalized weakness. Vital signs were significant for a temperature of 100.3 °F, heart rate of 113, respiratory rate of 20, blood pressure of 135/96, and oxygen saturation of 100% on 4 liters nasal cannula. On exam, he was a tired-appearing male in no acute distress, with well-healing abrasions to the chin and forehead. Pupils were equal and reactive with bilateral conjunctival injection on eye exam. Neurological exam was notable for intact strength and sensation with no focal neurological deficits. He was alert and oriented and followed commands. A laceration was noted on the side of the tongue. Lab work was obtained, and the patient was sent for CT of the head and cervical spine.

Upon return from CT, the patient had a witnessed seizure lasting one minute which terminated with administration of lorazepam. After the seizure, the patient was extremely agitated, bucking in the stretcher and pulling at his intravenous (IV) access, followed by a period of somnolence with gurgling respirations. He was intubated for airway protection and sedated with propofol.

On review of his CT scan following intubation, several small, peripherally calcified lesions were noted by the EM clinicians per radiology; these findings were most consistent with cystic infection of the brain, likely neurocysticercosis.

## Significant findings

In our patient, two lesions were most notable on CT in the frontal and occipital lobes. The lesion in the left frontal lobe (blue circle) was an approximately 1.5 centimeter (cm) rounded area with rim enhancement and surrounding hypodensity, consistent with vasogenic edema. A similar sized low-density area in the left occipital lobe (red circle) was noted, with increased peripheral density at the 3 o’clock position representing calcification. There were no areas of apparent hemorrhage or midline shift. The final radiology report concluded there were multiple cystic lesions, one with surrounding vasogenic edema in the left frontal lobe.

## Patient course

The patient was admitted to the intensive care unit under the care of neurology and infectious disease and treated with 10 milligrams (mg) IV dexamethasone and 4 grams (g) levetiracetam. He was extubated the following day. Twenty-four-hour video electroencephalogram (EEG) demonstrated no additional seizure activity. After he was extubated, the patient provided additional history that he had emigrated from Central America 5 years ago, and that he also had been hospitalized at a different facility for seizures 7 months ago. Records reviewed by the inpatient team revealed a previous diagnosis of neurocysticercosis for which the patient was treated with albendazole, but that he never picked up his outpatient prescription upon discharge. Polymerase chain reaction (PCR) testing was obtained (in lieu of enzyme linked blotting due to its availability), screening for tuberculosis (TB), strongyloides and human immunodeficiency virus (HIV) were negative, and an MRI redemonstrated three cystic lesions with ring enhancement. Infectious disease concluded the patient was likely in the degenerative phase of his disease and recommended treatment with albendazole and praziquantel. The patient continued to improve throughout his hospitalization and was discharged in good condition on levetiracetam to be taken for two years and a ten day steroid taper.

## Discussion

Neurocysticercosis predominantly affects large swaths of Central and South America, Asia, and Africa. It remains one of the most preventable causes of epilepsy worldwide, affecting 50 million people, primarily those with low socioeconomic status and in rural communities. The instance in the U.S. remains less than 2%, and therefore is infrequently considered in the differential diagnosis for etiology of new-onset seizure. In areas with high immigrant populations from endemic regions, this may represent an unrecognized but significant portion of patients presenting to the ED.^5^

Imaging findings in neurocysticercosis will vary based on the stage of the disease. The most common modalities include CT and MRI of the brain. Parenchymal neurocysticercosis proceeds through active stages: vesicular, colloidal vesicular, and granular nodular, followed by the inactive calcified stage.^6^ Cysts in the vesicular stage begin with thin, non-rim enhancing walls and may contain a central scolex, which subsequently disappears in the colloidal phase, while the cyst wall becomes thicker and more enhanced with surrounding edema due to degeneration of the cyst. In the granular stage, the cyst continues to shrink, and the surrounding brain develops gliosis resulting in thick rim enhancement. The final stage demonstrates near total calcification of the cyst with no surrounding edema. CT acts as the ideal screening tool for identifying cystic disease, particularly in the calcified stage. MRI provides more detailed images of active parenchymal stages, as well as nonparenchymal disease within the brainstem, subarachnoid, and intraventricular regions.^7^

The role of the ED clinician in managing neurocysticercosis centers largely on treatment of symptoms and maintaining the airway and breathing of the seizing patient. In our case, this involved airway security, anti-epileptic treatment, as well as recognizing the need for neuroimaging in a patient with unclear history of epilepsy. While CT is a less-expensive, rapid technique for identifying cystic lesions in the brain parenchyma, the differential diagnosis contains several other potential etiologies such as toxoplasmosis, echinococcus, and tuberculosis that may have similar appearances on CT. Further workup with MRI can clinch the diagnosis of NCC if a scolex is identified within the cyst, (a pathognomonic finding) as well as identify disease outside of the parenchyma. When combined with serologic testing and enzyme-linked blotting, sensitivity of these findings is near 100%. The Infectious Disease Society of America (IDSA) recommends treating any cystic infections of the brain resulting in seizures, regardless of etiology, with antiepileptics such as benzodiazepines or levetiracetam. Consensus treatment guidelines also recommend steroids, namely dexamethasone, if there is evidence of edema on imaging. While there is varying evidence regarding optimal dosing of dexamethasone, the IDSA recommends 0.1mg per kilogram (kg) per day. These treatments can be initiated in the ED with only the CT findings, such as the ones identified in our patient. If further workup confirms neurocysticercosis, consensus guidelines recommend treatment with seven to fourteen days of albendazole (15mg per kg per day) and praziquantel (50mg per kg per day) for viable cysts and certain cases of degenerating cysts. Antiepileptics and steroids, but not antiparasitics, are recommended in calcified disease.^8^, ^9^

Our case highlights the importance of considering this diagnosis in U.S.-based emergency departments with high concentrations of patients from endemic environments. Clinicians who note classic findings on neuroimaging can implement appropriate therapy, and bridge patients to neurology and infectious disease. Neurocysticercosis remains one of the most common preventable causes of seizures, and its recognition in the ED represents a significant opportunity for intervention and overall quality of life improvement for those affected.

## Supplementary Information




