# Supplementary figures and images for: Epilepsy Caused by Neurocysticercosis: A Case Report
Source: J Educ Teach Emerg Med. 2023 Jan 31;8(1):V14–7. doi: 10.21980/J81P96 (PMC10332771; doi:10.21980/J81P96)

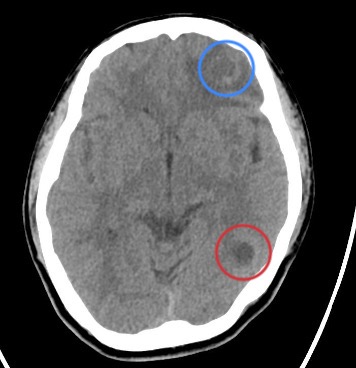

Supplement: Supplementary file 1 [file jetem-8-1-v14-supp1.jpg]

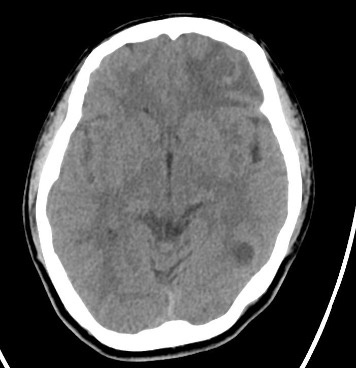

Supplement: Supplementary file 2 [file jetem-8-1-v14-supp2.jpg]
